# Mediastinal drain-induced bronchoperitoneal fistula following esophageal perforation: a case report

**DOI:** 10.1093/jscr/rjag181

**Published:** 2026-03-22

**Authors:** Naoki Ishimaru, Takashi Tagami, Kazuya Niwa, Kohei Takayasu, Kosuke Tobita

**Affiliations:** Department of Surgery and Emergency Medicine, Suwa Central Hospital, 4300 Tamagawa, Chino, Nagano 391-8503, Japan; Department of Emergency and Disaster Medicine, The Jikei University School of Medicine, 3-25-8 Nishi-Shimbashi, Minato-ku, Tokyo 105-8461, Japan; Department of Surgery, Suwa Central Hospital, 4300 Tamagawa, Chino, Nagano 391-8503, Japan; Department of Surgery, Suwa Central Hospital, 4300 Tamagawa, Chino, Nagano 391-8503, Japan; Department of Surgery, Suwa Central Hospital, 4300 Tamagawa, Chino, Nagano 391-8503, Japan

**Keywords:** bronchoperitoneal fistula, complications, esophageal perforation, mediastinal drain

## Abstract

A bronchoperitoneal fistula is a rare complication that is usually associated with intra-abdominal abscesses. Drain-induced bronchoperitoneal fistula following mediastinal drainage for esophageal perforation has not been previously reported. A 76-year-old man with peritoneal metastasis from advanced gastric cancer developed an esophageal perforation during endoscopic balloon dilation for malignant stricture. Since severe malignant esophageal induration precluded secure endoscopic stent deployment, emergency laparotomy was performed as a palliative strategy, including feeding jejunostomy and mediastinal drain placement. Five weeks postoperatively, computed tomography and contrast studies demonstrated migration of the mediastinal drain with a direct fistulous communication between the bronchus and peritoneal cavity. Prompt repositioning of the drain prevented severe respiratory complications; however, the patient died 2 months later owing to progression of the underlying malignancy. This case highlights that prolonged mediastinal drainage could very rarely result in serious iatrogenic bronchoperitoneal fistula, underscoring the importance of careful monitoring during long-term drainage.

## Introduction

Esophageal perforation is a serious condition with reported mortality rates of 12%–13% [1]. It can result from spontaneous rupture, foreign body ingestion, and iatrogenic injury. Prompt diagnosis and treatment are essential, with management centered on source control through primary repair, endoscopic stenting, or surgical diversion/exclusion, combined with adequate drainage [2, 3]. Although drainage is indispensable for controlling mediastinal contamination and fluid collections, prolonged or inadequate drain management may rarely cause serious complications, including infection, bowel obstruction, or iatrogenic fistula formation [4]. Here, we present an extremely rare case in which a mediastinal drain, placed to treat esophageal perforation, resulted in bronchoperitoneal fistula development.

## Case report

A 76-year-old man presented with a 2-day history of general malaise and difficulty swallowing. He had a history of distal gastrectomy for gastric cancer. Ten months prior, he underwent total gastrectomy for remnant gastric cancer. Pathological examination revealed a tumor-node-metastasis stage of pT4b, N2, M0, and stage IIIB (AJCC 8th edition). The resection margin was positive, and adjuvant chemotherapy was declined. Five months prior to admission, peritoneal carcinomatosis obstructed the descending colon, necessitating colostomy creation. Two months before admission, esophageal stenosis was treated with endoscopic balloon dilation.

**Figure 1:**
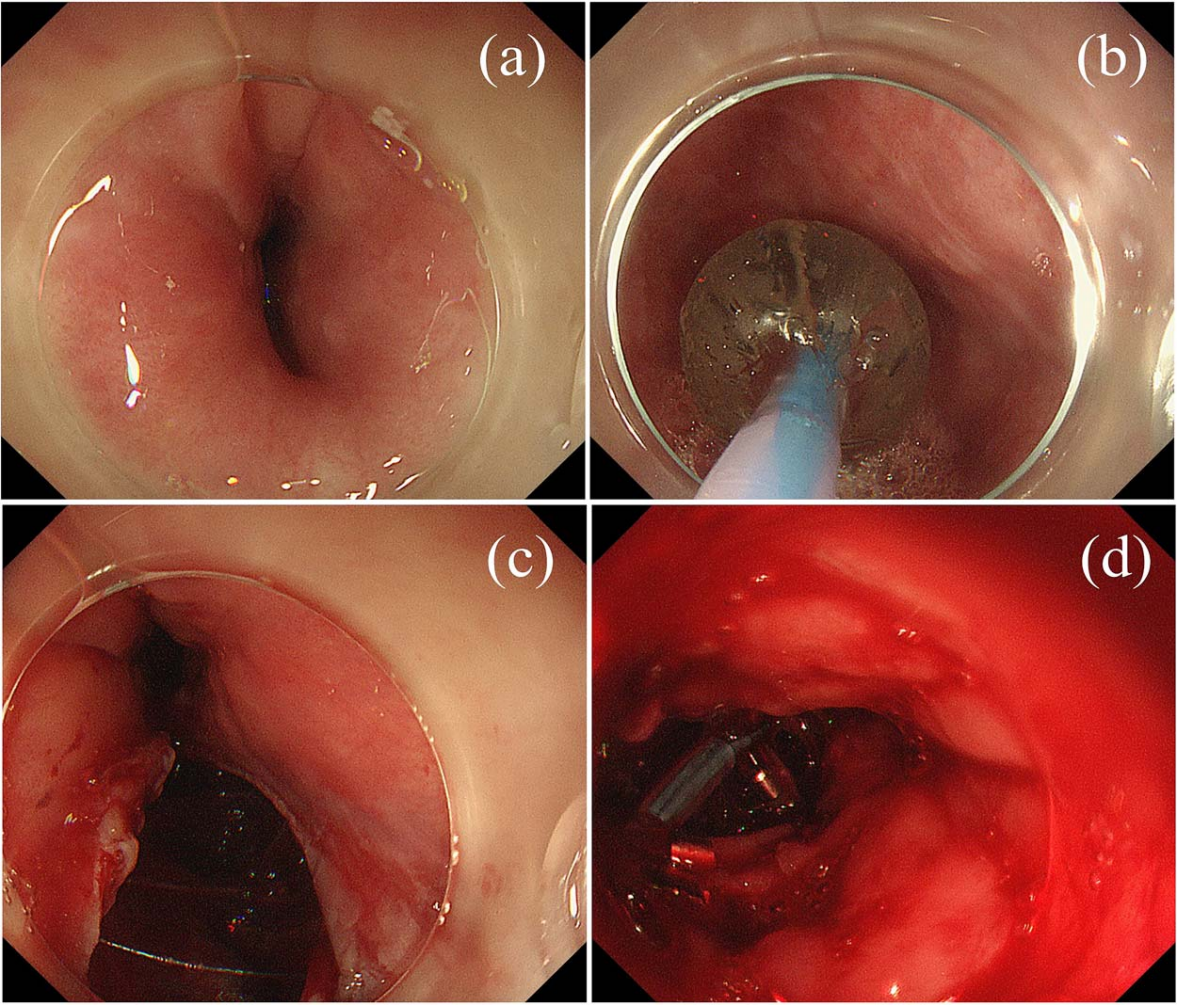
Endoscopic findings. (a) Upper gastrointestinal endoscopy showing esophageal stricture. (b) Balloon dilation procedure. (c) Perforation observed on the left side of the esophagus during the second dilation. (d) Unsuccessful endoscopic closure attempt using metallic clips.

**Figure 2:**
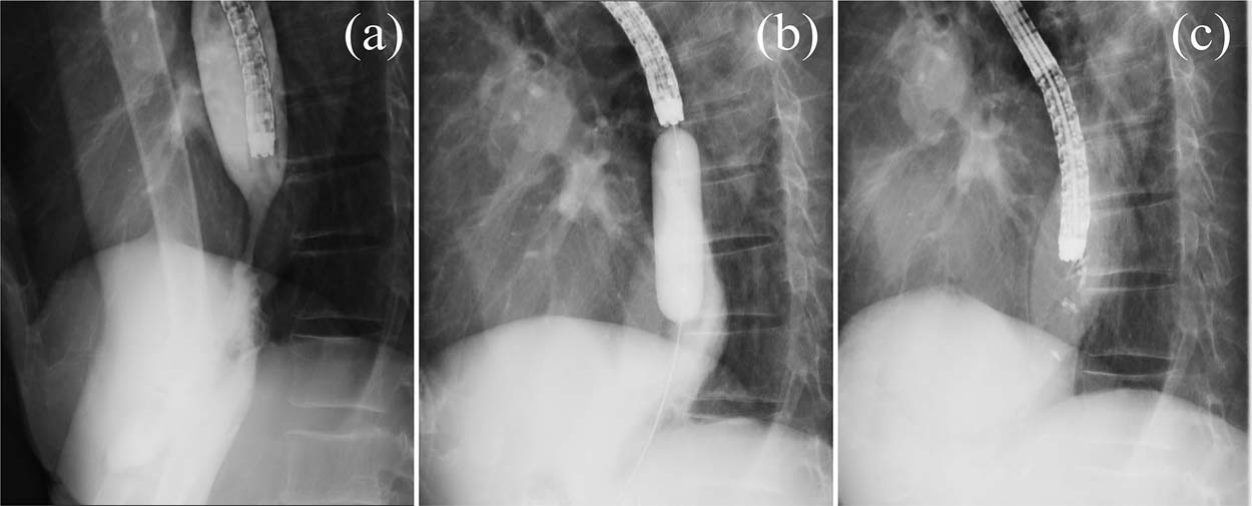
Esophagography. (a) Esophagography revealing esophageal stricture. (b) Balloon dilation procedure. (c) Attempted endoscopic closure of the esophageal perforation with clips.

Upon presentation, a chest and abdomen computed tomography (CT) scan revealed esophageal stenosis secondary to peritoneal dissemination. An upper gastrointestinal endoscopy confirmed the obstruction (Figs 1a and 2a). Balloon dilation (16.5 mm for 3 min) was performed (Figs 1b and 2b). During the second dilatation, a perforation occurred on the left esophagus wall (Fig. 1c). Endoscopic closure with clips was unsuccessful (Figs 1d and 2c), necessitating an emergency laparotomy. Endoscopic stenting was not performed due to severe esophageal induration and malignant stenosis, which precluded stable stent deployment and reliable sealing. The esophageal tissue was markedly indurated and unsuitable for primary repair. Consequently, a closed suction catheter was placed along the left esophageal wall in the mediastinum, and a feeding jejunostomy tube was inserted.

**Figure 3:**
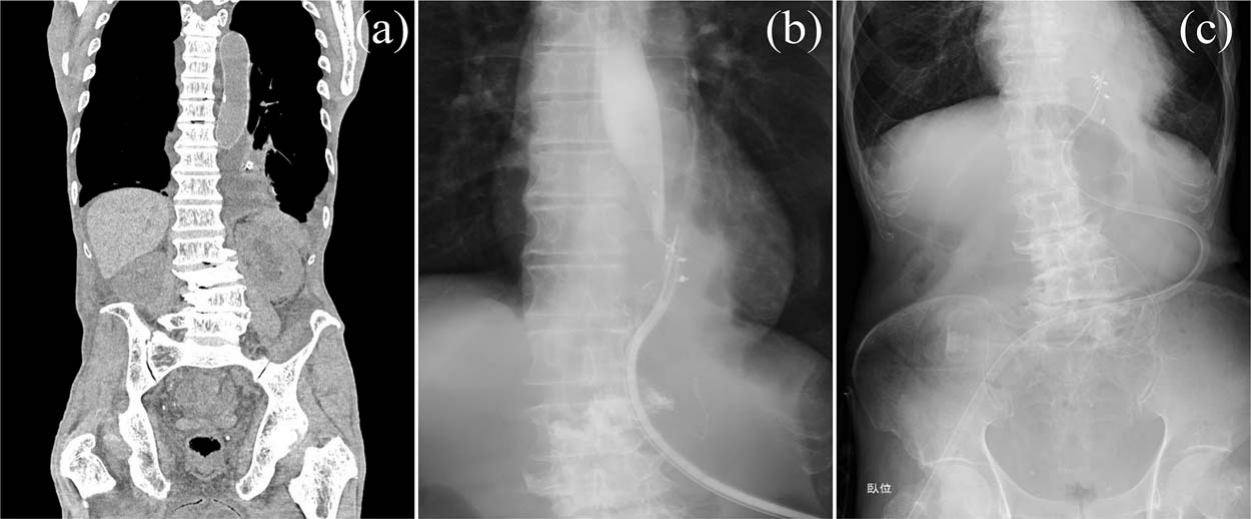
Postoperative imaging and monitoring. (a) Chest and abdominal CT scan demonstrating mediastinal abscess formation. (b) Contrast study through drainage tube revealing communication with the esophagus. (c) Chest radiographs monitoring pulmonary status and drain positioning.

**Figure 4:**
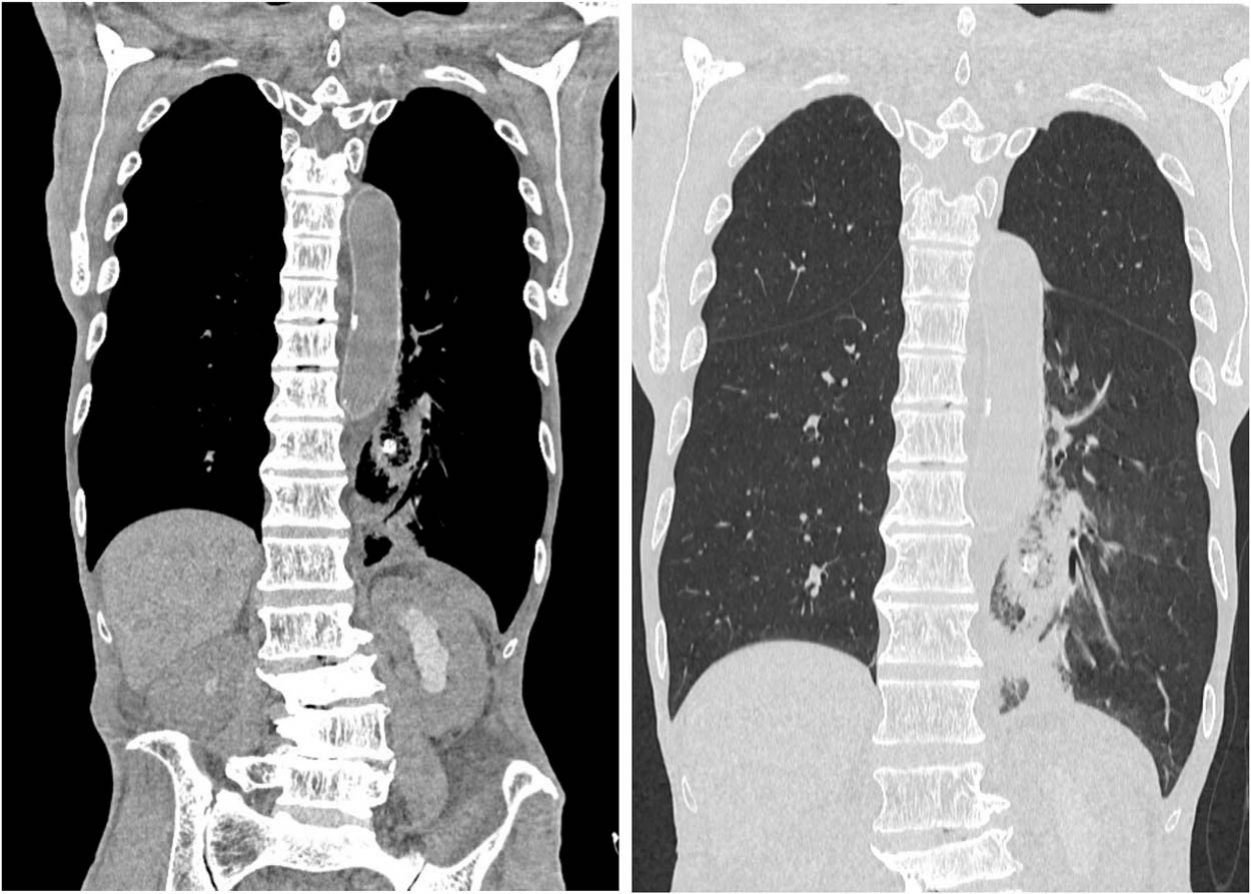
Postoperative drain migration. Chest and abdominal CT scan showing drain tip migration into the thoracic cavity.

**Figure 5:**
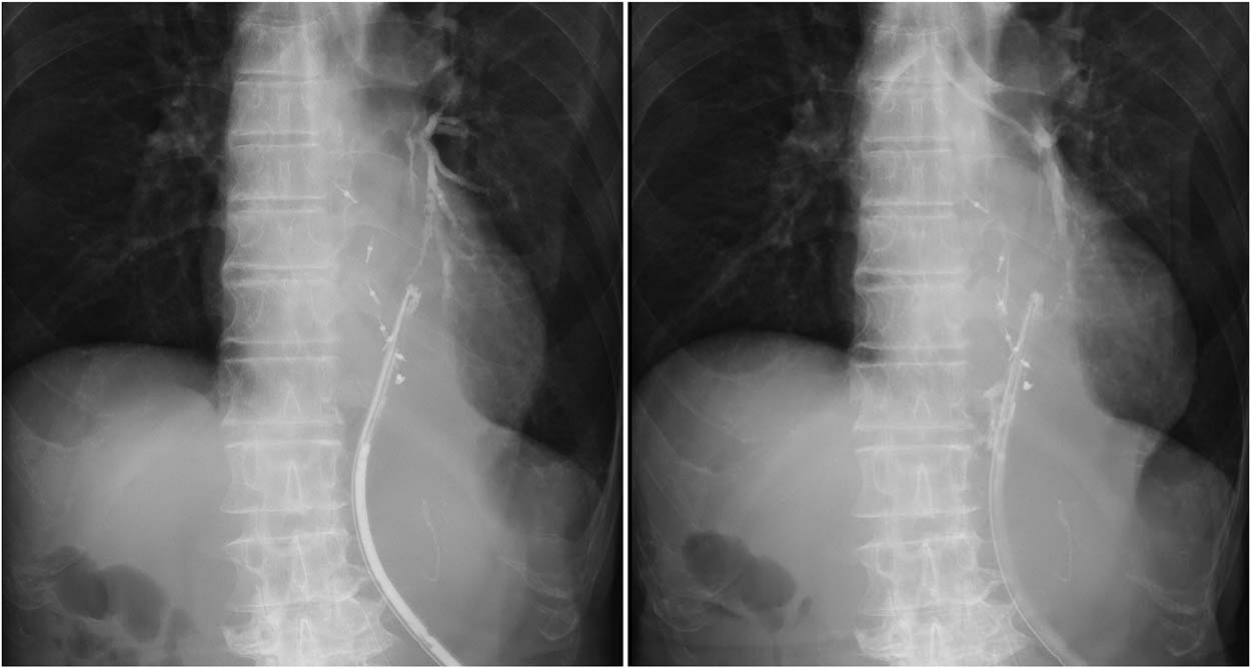
Bronchoperitoneal fistula formation. Contrast study through drainage tube demonstrating a fistula between the bronchus and peritoneal cavity.

**Figure 6:**
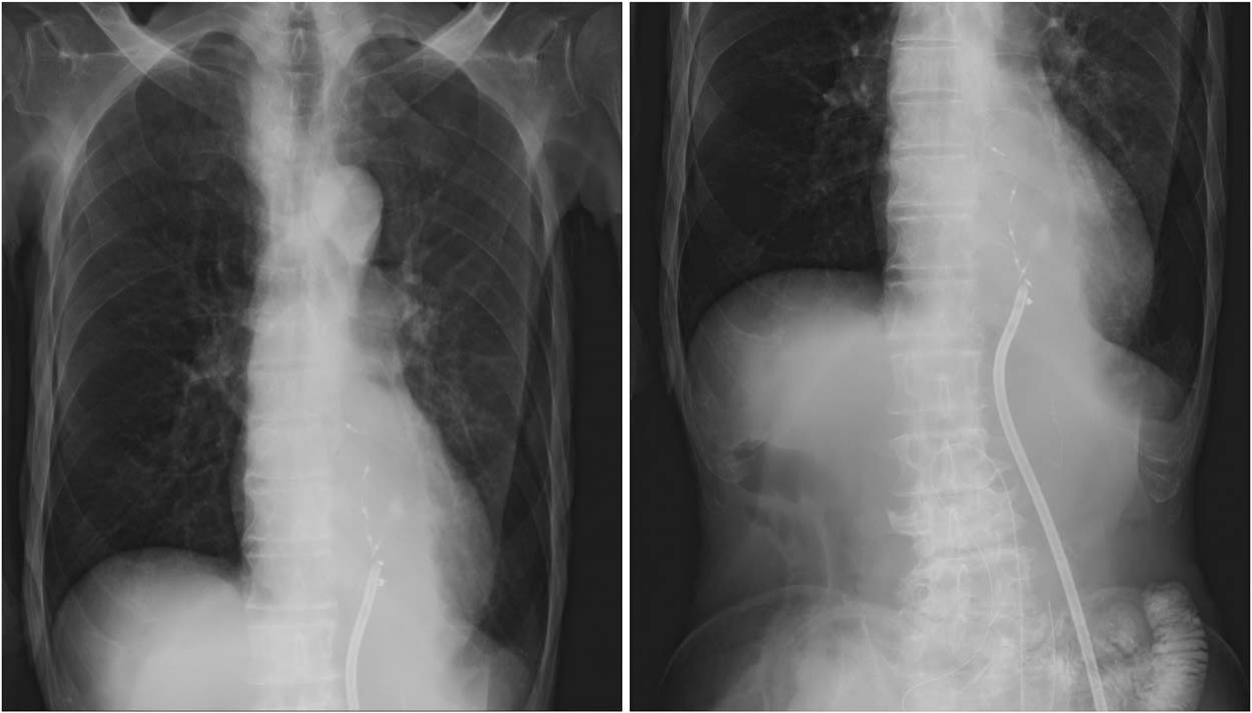
Drain repositioning. Chest radiograph and contrast study showing drain tip repositioning into the mediastinum.

One week after surgery, a chest and abdomen CT scan revealed the drain in the mediastinum with an abscess (Fig. 3a). Two weeks postoperatively, contrast study revealed esophageal communication (Fig. 3b). By the third week, contrast studies showed no further esophageal communication. Although leakage resolved, mediastinal abscess drainage persisted; the drain was left in place for source control, as premature removal risked uncontrolled mediastinal infection given advanced malignancy and poor tissue healing. Follow-up imaging showed stable drain positioning and abscess size. The patient remained clinically stable without fever, respiratory symptoms, or changes in drain output through the third postoperative week (Fig. 3c). Given this stable course, imaging intervals were extended. At 5 weeks postoperatively, routine surveillance CT was performed and revealed that the drain tip had migrated into the thoracic cavity (Fig. 4). A contrast study through the drainage tube revealed bronchoperitoneal fistula formation, with the drain creating a direct communication between the bronchus and peritoneal cavity (Fig. 5). The drain tip was repositioned back into the mediastinum (Fig. 6). Although no air leak was detected from the drain, a small amount of purulent drainage was observed; the drain tube was left in place.

The patient’s respiratory status remained stable, without signs of air leaks. Additional stenting to close the fistula was not pursued because respiratory status remained stable after drain repositioning and overall prognosis was limited by progressive malignancy. However, peritoneal dissemination and cancerous ascites progressively worsened. Despite supportive care, the patient’s condition deteriorated and expired ~2 months after the bronchoperitoneal fistula onset.

## Discussion

This report describes a rare case of bronchoperitoneal fistula formation associated with mediastinal drain migration into the thoracic cavity during postoperative care for esophageal perforation. Bronchoperitoneal fistula is typically associated with subphrenic or intra-abdominal abscesses; drain-induced cases are exceptional [4].

In the present case, severe esophageal induration and malignant stenosis precluded durable closure by stenting or primary repair. Therefore, management focused on source control and enteral nutrition given the advanced malignancy.

Al Khaldi *et al.* reported complications associated with intraperitoneal drains including infection, bleeding, bowel injury, intestinal obstruction, and difficulty in removal [5]. However, bronchoperitoneal fistula formation remains exceedingly rare. Most cases of bronchoperitoneal fistulas arise from intra-abdominal abscesses that penetrate the diaphragm. Documented causes include iatrogenic injuries during biliary surgery [6, 7], liver abscesses [8], complications of sleeve gastrectomy [9], and anastomotic leaks after colorectal cancer resection [10]. Only one previous case of bronchoperitoneal fistula caused by an intraperitoneal drain has been reported, involving a retained drain following abdominal surgery for a gunshot wound [4].

Although delayed drain migration likely contributed to fistula formation in this case, attributing causation to a single factor alone is challenging. Bronchoperitoneal fistulas are known to develop via inflammatory tract formation across the diaphragm, secondary to conditions such as subphrenic abscess or intra-abdominal infection [8–10]. In the present case, multiple factors coexisted, including persistent mediastinal inflammation following esophageal perforation, tissue fragility associated with advanced malignancy, and impaired wound healing due to peritoneal dissemination. These background factors likely created a predisposing substrate for fistula formation, upon which drain migration may have acted as a mechanical trigger. This case serves as a cautionary reminder that long-term mediastinal drain placement can result in rare but severe iatrogenic complications, particularly in patients with compromised healing capacity secondary to infection, inflammation, or malignancy.

Once the bronchoperitoneal fistula was identified, repositioning of the drain stabilized the clinical course. Further endoscopic or airway interventions were not added because there was no persistent clinical deterioration and goals of care remained palliative.

Bronchoperitoneal fistulas are known to cause complications, such as pneumonia, acute respiratory distress syndrome, and sepsis [4, 6, 11]. Airflow through the fistula into the abdominal cavity, along with increased intra-abdominal pressure, can impair wound healing and lead to anastomotic leakage [10]. In our case, major respiratory complications and abdominal air inflow were avoided, likely due to the timely repositioning of the drain and narrowing of the communication pathway caused by peritoneal seeding.

Clinicians should be vigilant for potential complications when long-term drain placement is necessary. Regular imaging studies are essential to monitor drain positioning, particularly in cases of esophageal perforation where mediastinal drainage is unavoidable. When drainage is prolonged, periodic reassessment and awareness of rare drain-related complications are important.

This report has limitations as a single case and cannot define incidence or risk factors. Therapeutic options were limited by advanced malignancy. Nevertheless, it highlights the importance of careful drain management and awareness of rare complications.

## Conclusions

This case demonstrated that mediastinal drain migration into the thoracic cavity can result in bronchoperitoneal fistula. In patients requiring prolonged mediastinal drainage, careful attention to drain-related complications is warranted.

## Consent for publication

Written informed consent for publication of this case report and accompanying images was obtained from the patient. The original consent form in Japanese bearing the patient’s handwritten signature is available from the corresponding author on reasonable request.

## Data Availability

The data that support the findings of this study are available from the corresponding author upon reasonable request.
